# The Price of Gold in Chile

**DOI:** 10.1289/ehp.114-1570089

**Published:** 2006-09

**Authors:** Scott Fields

Straddling the Chilean–Argentine border high in the Andes, near a field of glaciers, lies an ore body that by mining industry estimates is large enough to yield 750,000 ounces of gold, 30 million ounces of silver, and 5,000 tons of tradable copper concentrate (typically consisting of 25–30% copper) every year for 20 years. Whether these valuable metals would stay in the ground, at least for the foreseeable future, or be extracted in an open pit mine is the topic of an ongoing tug-of-war between environmentalists and Canada’s Barrick Gold Corporation, the claim holder and one of the world’s largest gold miners.

Just about any new extractive industry proposal will attract protests, says Vincent Borg, Barrick Gold’s senior vice president of corporate communications. He says Pascua-Lama, as the proposed mine is called, has been a target of environmentalists’ wrath just because it’s the latest large mine on the drawing table. But the Pascua-Lama case includes a few unique twists: perhaps 5% of the metals lie under glaciers, the site sits on land in two countries, and as gold and silver mines go, it is at a high altitude, a little over 15,000 feet above sea level. Tremors from regional earthquakes also are no stranger to Huasco Valley. According to the USGS, the region saw 6.7-magnitude earthquakes in 2006 and 2002, while a 6.8-magnitude event occurred in 2003.

By now, hoops have been jumped through, most i’s dotted and t’s crossed, protests conducted, and appeals exhausted. Just about all of the major players on this issue, for and against, say that the mine will almost certainly open in the near future. However, some activists are grasping at a thin thread of hope that new appeals to Chilean regulatory agencies, as well as other legal maneuvers could halt the project.

What concerns me is that this particular mine has a very high resource value. That can drive people to assuming and making promises about a project that just realistically can’t be upheld.

— Jim Kuipers, Kuipers and Associates LLC

## Initial Concerns

Much of the initial attention on Pascua-Lama focused on the issue of the glaciers that cover a portion of the site’s deposits. To get at some of the metal deposits, Barrick Gold at one time planned to relocate parts of three of the smaller glaciers, called Toro 1, Toro 2, and Esperanza.

Melt from all of the glaciers—which developed during the last ice age, don’t fully renew each winter, and have already diminished by 50–70% over the last 20 years—feeds primary sources of water used by local farmers and other inhabitants. The prospect of destroying glaciers, especially ones that indigenous people depend on so heavily, drew a flurry of attention from local activists and environmental groups in Chile and Argentina and from abroad. But Borg argues that these three glaciers are so small compared to the surrounding glaciers that they contribute little to the Huasco Valley watershed—less than 0.5%, by Barrick Gold’s estimate.

All that said, the whole glacier issue is now moot anyway, according to Borg: “The glaciers will not be moved as part of the actual project that was approved.” That’s because new Chilean president Michelle Bachelet said during her campaign and after taking office that she wouldn’t allow the glaciers to be touched. So the deposits that currently lie under these glaciers will stay put, Borg says, even if the ice covering them should disappear naturally later in the project.

Despite the glaciers’ surviving the mine unscathed, local groups worry that, like problematic mines in locations elsewhere, this mine will contaminate local water supplies, says David Modersbach, an independent activist who lives in Rosario, Argentina. Water impacts are usually the most troublesome issue associated with all types of mining, but especially with hard-rock mines like Pascua-Lama.

## Potential Water Problems

The water problems associated with hard-rock mining are essentially twofold: those associated with exposing buried rock, and those associated with accidents involving chemicals such as cyanide, mercury, and ammonia, which modern hard-rock miners use to extract gold and silver from pulverized rock. According to the U.S. EPA, mines can also affect waterways in other, more subtle ways, by altering natural drainage patterns, impounding water, diverting streams, and a host of other practices.

Acid mine drainage forms when the sulfide minerals in which gold and silver hide mix with specialized bacteria, air, and water. These acidic waters also can leak from natural rock formations, although in vastly smaller quantities. When sulfides are churned up and exposed to air, then introduced to water, they produce sulfuric acid, a medium in which these bacteria thrive. The nurtured bacteria oxidize the minerals further, resulting in a chain reaction that can produce water acidic enough to dissolve iron tools.

Unconstrained acid mine drainage can damage ecosystems when it finds its way into waterways and groundwater alike. It can also pepper waterways with potentially toxic metals—such as arsenic, lead, cadmium, mercury, zinc, iron, copper, aluminum, manganese, and chromium—that the acidic water leaches from the rock it flows through. Acid mine drainage has cost hundreds of millions of dollars to remediate in the past two decades alone, according to EPA estimates. Ideally acid mine drainage is controlled, usually by sequestering the depleted ore (“tailings”) and waste rock behind dams and other enclosures designed to prevent water from flowing through the sulfide minerals.

The other hazard that hard-rock mines pose to waterways is inadvertent releases of cyanide. Like virtually all gold or silver mines nowadays, Pascua-Lama will use cyanide to strip minute flecks of gold and silver from ore. In the “vat leach” process planned for Pascua-Lama, ore is pulverized and poured into enormous vats. Sodium cyanide solution—the annual use of a typical mine is measured in hundreds of tons—is mixed with the pulverized ore. Cyanide trickling through the ore collects gold and silver particles, forming water-soluble gold– or silver–cyanide compounds. The tailings are deposited in vast pools and held in place by dams.

According to Borg, the Pascua-Lama project has included in its design significant facilities to minimize the possibility of surface and subterranean water coming in contact with waste rock as well as a comprehensive system of passive and active barriers to collect, store, treat, and reuse any water that does come in contact with the waste rock. This will ensure the quality of the water downstream of the project. The mine will also include state-of-the-art facilities for controlling acid mine drainage, for handling cyanide (including wide, well-constructed roads for cyanide transport vehicles), and for monitoring water quality.

But according to a U.S. government mining expert who is familiar with the Pascua-Lama site and who asked not to be identified, the altitude and terrain of the site present unique challenges. In more hospitable locales, even the most up-to-date acid mine drainage prevention technologies, tailings holdings facilities, and cyanide handling schemes have failed, he says. In the past three decades, several tailings dams around the world have collapsed, and many more have leaked leftover cyanide and trace heavy metals leached from the ore. In some cases, even when enclosures have worked well, acid mine drainage has appeared from unanticipated spots outside the enclosures.

For the Pascua-Lama operation, the source says, the company would need “great engineering” to prevent the environmental damage that hard-rock mines often inflict. Once the mine closes, he says, restoring the site will be especially difficult because ecosystems at such high altitudes are fragile and slow-growing.

Besides dam failure, there is the possibility of cyanide tanker trucks crashing into or near waterways, dumping hundreds of gallons of cyanide into bodies of water. It is the prospect of just this kind of spill that most worries local residents, especially farmers, says Antonia Fortt, an environmental engineer at the Santiago office of Oceana, a Washington, DC–based environmental group. “On the Chilean side they are going to build their roads for the trucks of the mine just next to the river. These trucks will carry not just explosives and other materials, but also cyanide. We have had accidents with cyanide before, here in Chile,” she says. “If we have a spill of cyanide, it would be just a disaster.”

Fortt also notes that the trip will be precarious because of the mine’s extreme elevation; trucks will be pummeled by seasonal high winds near the summit. Further, she says, the company’s plan to dump waste rock at the headwaters of the Estrecho River could be equally, albeit more gradually, damaging if the rocks start generating acid.

## The Question of Environmental Impact

Many modern mines in developed countries—including U.S. sites in states such as Montana, Idaho, and Alaska—are getting better at mitigating the environmental risks posed by mines, says Jim Kuipers, an independent mining consultant and mining facility inspector for Kuipers and Associates LLC of Butte, Montana. “In a case like Pascua-Lama it is more difficult to predict what will happen in that kind of circumstance,” he says. Further, he adds, “It is fair to say that the ability to control it may be much more difficult just because of the climate, the topography, and issues like that.”

A few other mines have operated, with mixed success, in similarly strenuous environments, Kuipers says, and learning from them will be critical in properly designing this mine.

Kuipers also voices concerns about the speed of the Pascua-Lama permitting process, which took just 18 months compared to an average of 4-plus years for a U.S. mine. He says Chile holds mining companies to much lower standards, explaining, “If this site were in the United States, I think it would warrant a much harder look from the permitting standpoint: a full-blown environmental impact statement, adequate opportunity for public opinion, not just from the opposition standpoint but from a technical standpoint. . . . You would want very competent agencies with experienced people looking at it. And even then I would expect you would still have problems with this mine, but hopefully you would have foreseen those problems and identified ways to deal with them. But in this case I don’t see any way they could have possibly done this, as quickly as it has been escorted through the permitting process.”

Adds Kuipers, “What concerns me is that this particular mine has a very high resource value. That can drive people to assuming and making promises about a project that just realistically can’t be upheld.”

Besides the mine’s state-of-the-art facilities, Borg says that if the quality of the water drops below Chilean and Argentine standards, the company will stop operations until the problem can be fixed. The catch, warns Joan Kuyek, national coordinator for the Ottawa-based watchdog group MiningWatch Canada, is that other than the mining company’s good word, there is not much to compel it to adhere to this agreement.

Pascua-Lama, like other mines in the region, doesn’t have to provide a financial guarantee that it will protect surrounding terrain or restore the site to pre-mine conditions. In most of the developed world, including the United States and Canada, mining companies must post reclamation bonds, typically for tens of millions of dollars, to ensure that a detailed and periodically updated mining closure plan is followed. Borg says a reclamation bond will not be posted for Pascua-Lama; instead, he says, “The most significant aspects of the closure requirements will be built into the project from the beginning of construction.”

## A Test Case

Many of the activists who oppose this mine say that they consider Pascua-Lama—for which 75% of the deposits lie in Chile and 25% lie in Argentina—a test case for future mines in pristine environments in the Andes in Argentina and Chile and on the long border that divides them. The Treaty of Mining Integration between the countries allowing such dual-country operations was ratified in 2000 and signed into law in 2004.

On 13 June 2006 the Chilean National Environmental Commission agreed to endorse 2 of 46 complaints filed against the decision to allow the Pascua-Lama project to proceed. A week later, a Chilean court returned two of Barrick Gold’s Pascua Lama mining leases to a prospector who had agreed to sell them but had never received full payment from the company. The leases are listed as Barrick’s property in a 2004 protocol to the Treaty of Mining Integration; there is a slim chance that the recent ruling would therefore invalidate this protocol, according to an analysis published 26 June 2006 in the Chilean newspaper *El Mostrador*.

In the meantime, barring any further legal action, the company plans to begin construction at Pascua-Lama in September 2006, with production slated to begin three years later. At this point activists are not optimistic about the future of the region. “If everything goes okay with this one, the gold companies could start to exploit all of the Andes to the south,” says Fortt. “It’s very likely that all other further projects could be approved for them, and they could get all of their environmental permits. If so, we don’t think we will be able to stop other companies from coming. Through all the valley, agriculture has been developed. If they are allowed to destroy the valley—which is so pure—what keeps them from doing it anywhere else?”

## Figures and Tables

**Figure:**
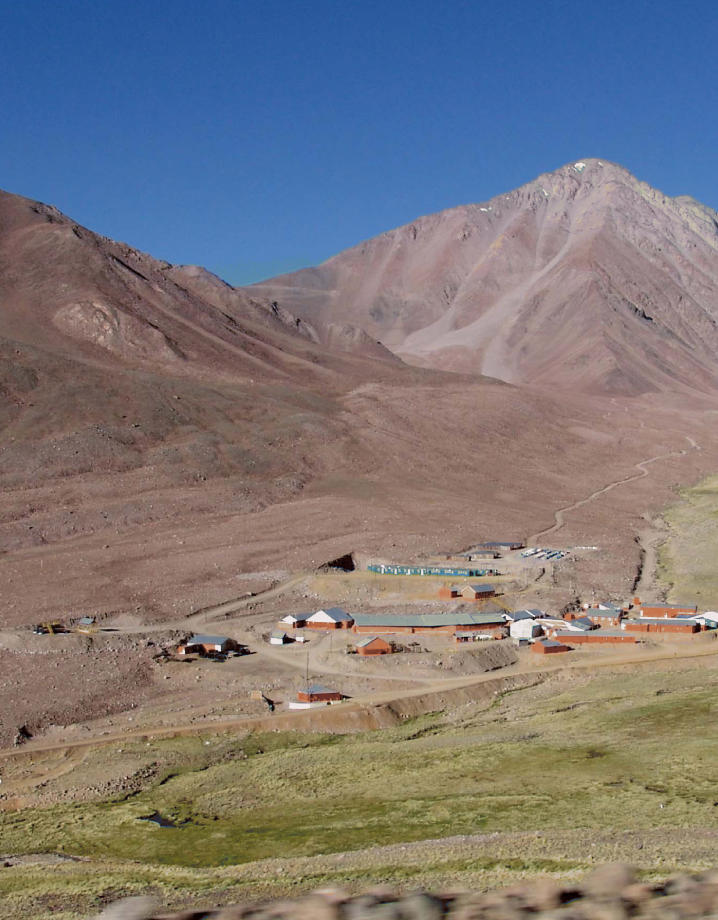


**Figure:**
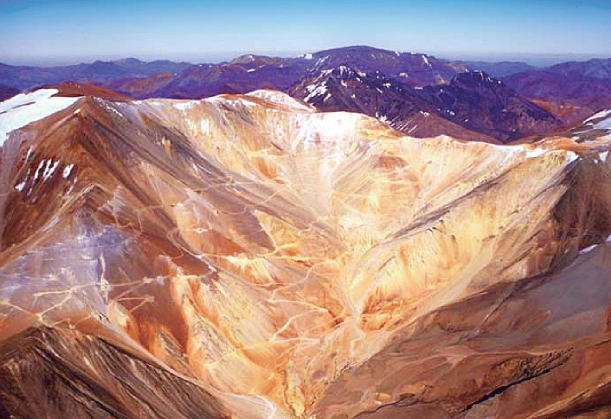
Source of gold—and contention Glaciers along the Chilean–Argentine border (above) narrowly missed being relocated to permit open pit gold mining at the Pascua-Lama site. At left is a mining exploration camp in the same area. Environmentalists continue to oppose the proposed mine.

